# Supplementary material for: The lived experience of chronic headache: a systematic review and synthesis of the qualitative literature
Source: BMJ Open. 2017 Dec 15;7(12):e019929. doi: 10.1136/bmjopen-2017-019929 (PMC5778309; doi:10.1136/bmjopen-2017-019929)
Supplement: Supplementary file 1 [file bmjopen-2017-019929supp001.pdf]

The lived experience of chronic headache: A systematic review and synthesis of the qualitative literature: Supplementary Material

Appendix 1 Search example

Medline Search<br>exp Headache/ or exp Headache Disorders, Secondary/ or exp Cluster Headache/ or exp Headache Disorders, Primary/ or exp Tension-Type Headache/ or exp Headache Disorders/exp Migraine Disorders/(headache* or migraine*).ti,ab.((rebound or transformed) adj5 (headache* or migraine*)).ti,ab.<br>exp Qualitative Research/exp Anthropology, Cultural/exp Feminism/exp Focus Groups/exp Grounded Theory/(interview* adj5 patient*).ti,ab.exp Narration/exp Personal Construct Theory/ exp Psychoanalysis/content analys*.ti,ab.thematic analys*.ti,ab.qualitative research.ti,ab.qualitative method*.ti,ab.qualitative stud*.ti,ab.constant comparison.ti,ab.content analys*.ti,ab.descriptive stud*.ti,ab.discourse.ti,ab. ethnography.ti,ab.feminist.ti,ab.focus group*.ti,ab.grounded theory.ti,ab. hermeneutic*.ti,ab.interpretive.ti,ab.lived experience.ti,ab.narrative.ti,ab. naturalistic.ti,ab.participant observation.ti,ab.phenomenolog*.ti,ab.personal construct theory.ti,ab.psychoanaly*.ti,ab.<br>limit 38 to yr="1988 -Current"<br>limit 39 to humans

Appendix 2. Excluded papers

Title	Headache type	Definition of chronicity	Comments
Leiper, D. A., et al. (2006) "Experiences and perceptions of people with headache: a qualitative study." BMC family practice 7: 27<br><br>N= 17<br>9M: 8F<br>Semi structured interviews<br><br>Framework approach with thematic analysis	Classified as having chronic head pain.<br>Migraine, tension type or mixed<br>(Two people self-reported their head pain as related to sinus and high blood pressure respectively.)	Chronic pain was defined as pain or discomfort, present either all the time, or on and off, which had persisted for 3 months or longer.<br>5 of the 17 self-report as having headaches as either daily or 'daily to weekly'.	Interviewees were sampled from a population based study investigating the prevalence and natural history of chronic pain.<br>Chronic pain was taken from The International Association for the Study of Pain taxonomy in 1986 this timing is often used in Chronic pain literature which does not directly map into the IHS definitions (26).<br>Data on CH participants not reported separately
Peters, M., et al. (2003). "Patients' decision-making for migraine and chronic daily headache management. A qualitative study." Cephalalgia : an international journal of headache 23(8): 833-841<br><br>N=13<br>4M:9F<br>Semi structured interviews<br>Grounded theory	Migraine and chronic daily headache	5 of the 13 had a diagnosis of chronic daily headache, data not reported separately	Data on CH participants not reported separately
Peters, M., et al. (2004). "Migraine and chronic daily headache management: a qualitative study of patients' perceptions." Scandinavian Journal of Caring Sciences 18(3): 294-303<br><br>N=13<br>4M:9F<br>Semi structured interviews<br>Grounded theory	Migraine and chronic daily headache	5 of the 13 had a diagnosis of chronic daily headache	Data on CH participants not reported separately
Peters, M., et al. (2005). "The patients' perceptions of migraine and chronic daily headache: a qualitative study." The Journal of Headache and Pain 6(1): 40-47<br><br>N=13<br>4M:9F	Migraine and chronic daily headache	5 of the 13 had a diagnosis of chronic daily headache,	Data on CH participants not reported separately

Semi structured interviews<br>Grounded theory			
Darghouth, S., et al. (2006). "Painful languages of the body: experiences of headache among women in two Peruvian communities." Culture, Medicine and Psychiatry 30(3): 271-297.<br><br>N=10<br>0M:10F<br>Structured open ended in-depth interviews<br>Ethnographic oriented, content analysis	Headache is reported as a symptom rather than a diagnosis. No individual characteristics are reported. This study is orientated towards the ways in which narratives of headache come to recount personal and shared stories of distress and affliction.	Defined chronic headache as being at least twice a week for at least one year	Their definition seems to fit more with an episodic presentation over a long period of time with a highly individual context of war, poverty and migration. They gave no further information about individual participant's frequency of headache so we were unable to compare definitions.

Appendix 3. Reciprocal translation across 3rd order themes

**1a Headache as a driver of behaviour - Direct**				
**Aspects**	Tenhunen	Coeytaux	Lonardi	Jonsson
Prioritisation/ changes to lifestyle	'…had to prioritize daily activities according to what their headaches would permit.' Pg 400	required a degree of adjustment roughly in inverse proportion to the degree of functionality.'Pg 482		they could only manage the most important parts of life…'Pg 4<br>had to make compromises and could not have the life they would have liked to live.' Pg 5<br>changes to their lifestyle, such as changing daily routines and avoiding trigger factors… avoid stress. Pg 6
Work	'…performance in the workplace was affected by absences Pg 400			'They developed strategies to manage work …' Pg 5
Reduced social contact	'…headache attacks combined with fatigue were the main reasons for the reduction in social contacts.'Pg401			
Emotional reactions	'…emotional responses to their headaches included Frustration, stress, depression Pg 402			
Loss of control	'Reduced …ability to control how their headaches affected their lives.' Pg403		"The head affects everything else…~~it~~ Pg 1623	'They had to make adjustments…whereas on the other hand they did not want headaches to take over their lives entirely by adjusting too much.' Pg 5
Inability to plan	'Inability to plan because of headaches…' .Pg 403,404	'…difficulty in predicting the frequency and severity of their headaches. This uncertainty made planning difficult or resulted in last minute cancellations…' Pg 483	'Planning ahead is made impossible…'Pg 1622<br>"…I lost my freedom to plan what to do tomorrow…"Pg 1622	'Being unable to plan things was considered debilitating and limiting.' Pg 5
Measuring headaches		'… the pain diary provided a meaningful expression of their level of pain, but more importantly, allowed them to see improvement of which they might otherwise have been unaware.'Pg 483		
Testing numerous strategies				'They were searching for strategies to manage the headaches and tried almost anything that they believed might be effective, regardless of the

				costs in terms of money or effort.' Pg 6<br>'Sleep was also important. They were taking various measures to improve their sleep, e.g. going to sleep courses or taking sleeping pills.' Pg 6
Medication use				Taking medication because one has to, not because one chooses to'…they viewed themselves as forced to increase their medication use.' Pg 7<br>Always having the medication at hand. 'Having the medication at hand made them feel calm and secure. If they realized that they had forgotten the medicine, they became anxious and had feelings of panic…' Pg 7
**1b Headache as a driver- Indirect**				
**Aspects**	Tenhunen	Coeytaux	Lonardi	Jonsson
Financial (and work)	'impaired performance at work, frequent absences and inability to take on greater responsibilities' affect income and economic status Pg 400,401			'… headaches would affect more long-term factors such as wages and pensions and that they could eventually force them to choose a less demanding job or even early retirement. Pg 5
Loss of energy and concentration (and poor sleep)<br>Poor sleep (and analgesics)	Poor sleep led to fatigue, loss of energy and concentration during the day. '…Analgesics contributed to tiredness or loss of energy.'Pg 401<br>Taking analgesics at night affect sleep patterns. Pg 401			
Emotional reactions (and social)	'Social restrictions imposed by headaches lead to feelings of isolation and frustration.'Pg401		Social exclusion leads to losing personal value as a human resource, experiencing related feelings of exclusion, isolation and loneliness.' Pg 1623	'They were sad that they had to forego things like a social life, exercise, travel and hobbies.' Pg4

Social activities (and medication)	Social activities were affected by the need for analgesics as well as the headaches themselves Pg 402			
Increasing medication				Increased medication use during stressful periods of life. Pg 7<br>Perceptions about the link between increasing headaches and medication use. 'In their view they used more and more medication because they had more headaches not vice versa.' Pg 7<br>Some in denial others suspect that the medication may be causing the headaches. 3 quotes pg 8
Link with psychological factors				Participants also considered the relationship between headaches and psychological factors such as stress, fatigue and depression. Pg 6

2 The spectre of headache				
**Aspects**	Tenhunen	Coeytaux	Lonardi	Jonsson
Emotional responses	'Emotional responses…were sometimes in response to impairments…' such as guilt or feeling like a burden on others.Pg 403			Headaches threaten to ruin one's life and are unbearable. Some were afraid of the pain, afraid of the next attack.Pg 4<br>'Sometimes they sensed that other people were suspicious…This suspicion made them angry and sad.' Pg 6
Potentially affects future relationships	'Headaches affected the prospects of developing stable relationships.' Pg 403		'The 'passing' dilemma between absolute secrecy vs total disclosure of information about stigma and the possible outcome of choices in terms of risk ("discredited/discreditable") lead directly to the decision-making solution for those who have chronic headache and are deeply affected by the invisibility of the disease.'Pg 1625	
Perceived loss of control	'Lost control of medium and long term occupational strategies…'Pg403			'The participants viewed their acute medication as indispensable because they perceived it to be the only thing that was effective against their headaches. They believed that without the medication, the negative consequences of headaches would ruin their lives…they depended on the medication to maintain their current lifestyle.'Pg 4
Medication use and beliefs	'…Using analgesics in anticipation of pain to control the disruptive effects of headaches on social planning.'Pg 403			'They were reluctant to use prophylactic medication because they did not want to medicate daily….they were reluctant to add another medication…They were afraid of side effects.' Pg 6<br>Resignation: Nothing but the medication helps. 'The only thing that had really helped was the acute

				medication…For some their resignation meant that they treated themselves to the medication whenever they felt that they needed it, without feeling guilty.' Pg 6<br>Avoidance of tracking medication use. 'They were reluctant to think about how much medication they actually used and avoided acquiring a clear overview of their medication use…Some deliberately avoided keeping track of their medication use…' Pg 7
Meaningful symptom relief		'…an increase in the number of pain free days would be most meaningful to them.' '….participants indicated a desire for medication that would reliably relieve headache pain.'Pg483		
The search for meaning/explanations			'It is the search…for a rational explanation.'Pg 1623<br>' The representation of the 'disease dimension' comes to light when…the patient finds that a certain medical diagnosis is acceptable and coincides with his/her subjective perceptions.' Pg 1623<br>"…I'm scared that something uglier is there but there's nothing…my worry is always the same but when I see that all the tests are negative, then I wonder why I have a headache."Pg 1623	'They expressed a need to find out what was causing the headache…At times they worried that the headaches were a symptom of serious illness, such as a tumour or a stroke.' Pg 6

3 Strained relationships				
**Aspects**	Tenhunen	Coeytaux	Lonardi	Jonsson
Work	Some ‘negative attitudes’ when colleagues work life was affected.Pg 401			
Friends	‘…how their friends responded to their headaches…’ Pg402 Friends responded to analgesic use differently. 401Pg	‘…each group reported strained relationships resulting from the need to change or cancel plans because of headaches.’Pg 483		‘It was hard to disappoint others and be unable to participate.’Pg 5
Changed relationships	‘Almost all… reported changes in the way others perceived them.’ Seen as weak or put in a sick or dependent role.Pg401			
Stress on others	‘…headaches placed stress on relationships not only by affecting their own behaviour but also because of the confusion, frustration and fear experienced by their partners.'Pg 402			
Helped by others	‘…their colleagues who were usually understanding and supportive…’ Pg 401		The patient is fully accepted into the inner family.’ Sometimes associated with the family being hyper-protective and external risk and harm may be envisaged.Pg1623	
Devalued by others		‘Participants agreed that an important aspect of their headaches involved the tendency of others to discount their impact.’ Pg 493 ‘…they had been labelled unreliable or accused of malingering. Participants were particularly critical of healthcare professionals…’Pg483	‘…although the social representation of the disease is managed within the family it is nevertheless incomplete.’ ‘The patient is recognised as having problems due to a disease but this is a reason for constant and explicit under- evaluation. Possibly due to a shared stereotype, everyone has experienced headaches that are generally mild, periodical, and provisional, and go away after taking a tablet.’Pg 1624	‘Sometimes they sensed that other people were suspicious, presumably thinking that headaches were being used as an excuse.’ Pg 6

			‘…a person may risk being the victim of social death….experiencing expulsion from the productive world…’ ‘…the inner family network accepts the person as ill but the wider or external network refuses him/her: employers or colleagues can in fact stigmatize the person as unreliable, lazy ,listless while neighbours and friends tend to trivialise the condition. Pg 1625	

Appendix 4. Third order conceptual theme 1 **Headache –a driver of behaviour change**

**Aspects**	**Interpretations of: T= Tenhunen et al 2005, C=Coeytaux et al 2007, L=Lonardi 2007,J= Jonssson et al 2013**	**Quotes/supporting data**
**Directly**		
Prioritisation/changes to lifestyle	'…had to prioritize daily activities according to what their headaches would permit.'T Pg 400	" I need to list everything regarding the daily tasks. If an electricity bill is due tomorrow it's the first one on the list……and do as many things as possible depending on my headache"T Pg 400
	'Pain characterised as moderate allowed some degree of function but required a degree of adjustment roughly in inverse proportion to the degree of functionality.'C Pg 482	" I wouldn't be running, I won't be reading but I can get through the day – I can go to work." C Pg 482
	'Headaches were an extra burden… they could only manage the most important parts of life…' J Pg 4	"When I should be making decisions and thinking clearly, then I don't…it takes the focus away from what you are supposed to be thinking about." J Pg 4
	'Because of headaches, they had to make compromises and could not have the life they would have liked to live.' Pg 5	"My life is very handicapped…or limited…it is incredibly limited. Pg 5 J
	'They also made changes to their lifestyle, such as changing daily routines and avoiding trigger factors. An important issue was to avoid stress. J Pg 6	No data
Work	'…performance in the workplace was affected by absences T Pg 400	"I have to cancel meetings…reschedule meetings…I mean that loss of working hours influences productivity."T Pg400
	'They developed strategies to manage work despite headaches…' J Pg 5	…such as working in the evening…or planning projects so there were extra time buffers…'J Pg 5
Reduced social contact	'…headache attacks combined with fatigue were the main reasons for the reduction in social contacts.'T Pg401	"Social activities are quite rare in my life" T Pg 401
Emotional reactions	'…emotional responses to their headaches included Frustration, stress, depression T Pg 402	"Because my headache affects every area of my life…sometimes more sometimes less…I fell quite trapped…and frustrated and angry. Yes and sometimes depressed as well…"T Pg 402
Loss of control	'Reduced …ability to control how their headaches affected their lives.' T Pg403	"I don't have as much control over my life as my husband has…" "I have to do whatever my illness requires and allows me to do."T Pg 403/4<br>"The head affects everything else…it is years since I've worn perfume because nine mornings out of ten I wake up with nausea…" L Pg 1623

Inability to plan<br><br>Measuring headaches<br><br>Testing numerous strategies<br><br>Medication use	'The subjective representation of the disease, the 'illness dimension', is what people experience first through the painful symptoms…'L Pg 1623<br>'They had to make adjustments…whereas on the other hand they did not want headaches to take over their lives entirely by adjusting too much.' J Pg 5<br><br>'Inability to plan because of headaches…' .T Pg 403,404<br><br>'…difficulty in predicting the frequency and severity of their headaches. This uncertainty made planning difficult or resulted in last minute cancellations…' C Pg 483<br>'Planning ahead is made impossible…'L Pg 1622<br><br>'Being unable to plan things was considered debilitating and limiting.' J Pg 5<br><br>'… the pain diary provided a meaningful expression of their level of pain, but more importantly, allowed them to see improvement of which they might otherwise have been unaware.'C Pg 483<br><br>'They were searching for strategies to manage the headaches and tried almost anything that they believed might be effective, regardless of the costs in terms of money or effort.'J Pg 6<br>'Sleep was also important. They were taking various measures to improve their sleep, e.g. going to sleep courses or taking sleeping pills.' J Pg 6<br><br>Taking medication because one has to, not because one chooses to'…something they did because they had to…not because they chose to…During periods in life with increased headache frequency, they viewed themselves as forced to increase their medication use.' J Pg 7	"Having these headaches controls my life, although I refuse to let it, it does in many ways." J Pg 5<br><br>"So that I don't have to cancel anything I don't make very big plans." T Pg 403<br>"…you can never tell when you're going to be able to do things…except at the last minute."C Pg 483<br><br>"…I lost my freedom to plan what to do tomorrow…"L Pg 1622<br><br>"I was handicapped in a way… life went on as usual, but I could not plan things." J Pg 5<br><br>"… I think my diary was more helpful than anything to me ... looking at the whole month." "After I had a month laid out and I'm looking at all these inverse relationships and noticing how I'm doing - that was [helpful]." C Pg 483<br><br>"I have tried lots of treatments: have spent an incredible amount of money…"J Pg 6<br><br>No data.<br><br>"It's not really that I am dependent on the medication itself, it is just that I do not want to feel like this. I want to get rid of the headaches and eventually one gets a little bit desperate."J Pg 7<br><br>"I have always got this little box with tablets in my pocket…It is my security blanket." J Pg 7

	Always having the medication at hand. 'Having the medication at hand made them feel calm and secure. If they realized that they had forgotten the medicine, they became anxious and had feelings of panic…' J Pg 7	
**Indirectly** *(knock on effect)*<br>Financial (and work)	'impaired performance at work, frequent absences and inability to take on greater responsibilities' affect income and economic status T Pg 400,401<br>'They also thought that their headaches would affect more long-term factors such as wages and pensions and that they could eventually force them to choose a less demanding job or even early retirement. J Pg 5	"…I have to turn some offers down" T Pg 400<br>One participant had been made redundant another became unemployed T Pg 401<br>"As for investing in me at work, concerning both salary and things like that…It should not have any influence, but I think it does anyway…"J Pg 5
Loss of energy and concentration (and poor sleep)	Poor sleep led to fatigue, loss of energy and concentration during the day. '…Analgesics contributed to tiredness or loss of energy.'T Pg 401	"…somehow I don't feel very fresh…""…it's difficult to concentrate on reading…things don't seem to stay in my memory." "The pain killers that really help me also cause drowsiness." T Pg401
Poor sleep (and analgesics)	Taking analgesics at night affect sleep patterns. T Pg 401	"…I have to wake up several times a night to take some more medication and the regular awakenings are pretty disturbing" T Pg 401
Emotional reactions (and social)	'Social restrictions imposed by headaches lead to feelings of isolation and frustration.'T Pg401<br>Social exclusion leads to losing personal value as a human resource, experiencing related feelings of exclusion, isolation and loneliness.' L Pg 1623<br>'They were sad that they had to forego things like a social life, exercise, travel and hobbies.'J Pg4	"…I probably have isolated myself a bit."T Pg401<br>"I tend to isolate myself…I go into the darkness, close my shutters. I left them closed for years in the afternoon." L Pg 1623<br>No data
Social activities (and medication)	Social activities were affected by the need for analgesics as well as the headaches themselves T Pg402<br>Increased medication use during stressful periods of life. Pg 7 Perceptions about the link between increasing headaches and medication use. 'In their view they used more and more	"I want to … live as normal life as possible…go on holiday without taking tens of different pills with me." T Pg402<br>"Then there was a period when I had headaches every day again, constantly, and then I took tablets all the time…I just wanted something that would make it go away…"J Pg 7

Increasing medication<br><br><br><br><br>Link with psychological factors	medication because they had more headaches not vice versa.' J Pg 7<br><br>Participants also considered the relationship between headaches and psychological factors such as stress, fatigue and depression. J Pg 6	Some in denial others suspect that the medication may be causing the headaches. 3 quotes J pg 8<br><br>"It becomes more stressful when I start working and then I get headaches too." J Pg 6

Third order conceptual theme 2 **The spectre of headache**

**Aspects**	**Interpretations of: Tenhunen et al 2005, Coeytaux et al 2007, Lonardi 2007, Jonssson et al 2013**	**Quotes/supporting data**
Fear of future<br><br><br><br><br><br><br>Emotional responses<br><br><br><br><br><br><br><br>Potentially affects future relationships	"…fears focused mainly on the prospect of worsening pain and how they and their family would cope." Pg401<br>'Identity is harmed because of the lack of possible viable perspectives.'L Pg1622<br>'Sometimes they even avoided making appointments because they dreaded having to cancel them.' J Pg 5<br><br>'Emotional responses…were sometimes in response to impairments…' such as guilt or feeling like a burden on others. T Pg402<br>Headaches threaten to ruin one's life and are unbearable. Some were afraid of the pain, afraid of the next attack.J Pg 4<br>'Sometimes they sensed that other people were suspicious…This suspicion made them angry and sad.' J Pg 6<br><br>'Headaches affected the prospects of developing stable relationships.' T Pg403<br>'The 'passing' dilemma between absolute secrecy vs total disclosure of information about stigma and the possible outcome of choices in terms of risk ("discredited/discreditable") lead directly to the decision-making solution for those who have chronic headache and are deeply affected by the invisibility of the disease.'L Pg 1625	"I'm a bit afraid of the pain… and afraid of how my family is going to cope with it if it gets worse." T Pg 402<br>"I don't know when the headache will come either, so I start panicking about where I'll be feeling pain next…" L Pg1623<br>No data<br><br><br>"Sometimes I also feel like a trouble to the others. Dependent on everybody." " I think that I'm causing a lot of extra work for my wife…"T Pg 402<br>" It is a terrible thing. I can't do anything… there are suicidal thoughts. It is so awful" J Pg 4<br><br>No data<br><br>"…I actually find it really difficult to find a guy who would understand my headache problem…" T Pg 403<br>"I pretend not to have it because in certain places, for example at work, nobody helps you…But how do you make them understand what it means to have a migraine 24h day… I don't say anything, otherwise I would be penalised…"L Pg 1625<br>"My friends go and I stay at home…nevertheless they understand my problem…they know I tell the truth…" L Pg 1625

Perceived loss of control	'Lost control of medium and long term occupational strategies…' T Pg403<br>'The participants viewed their acute medication as indispensable because they perceived it to be the only thing that was effective against their headaches. They believed that without the medication, the negative consequences of headaches would ruin their lives…they depended on the medication to maintain their current lifestyle.'J Pg 4	"I was thinking of extending our shop…… I didn't want to bite off more than I could chew…because of my headache…"T Pg403<br>"These triptans are the only thing that I have found that really helps, so that I can live my life and do what I want to… if it stops or if I'm not allowed to take it any more…Just thinking about it makes me very nervous."J Pg 4
Medication use/beliefs	'…Using analgesics in anticipation of pain to control the disruptive effects of headaches on social planning.'T Pg 403<br>'They were reluctant to use prophylactic medication because they did not want to medicate daily….they were reluctant to add another medication…They were afraid of side effects.'J Pg 6<br>Resignation: Nothing but the medication helps. 'The only thing that had really helped was the acute medication…For some their resignation meant that they treated themselves to the medication whenever they felt that they needed it, without feeling guilty.' J Pg 6<br>Avoidance of tracking medication use. 'They were reluctant to think about how much medication they actually used and avoided acquiring a clear overview of their medication use…Some deliberately avoided keeping track of their medication use…' J Pg 7	" If I have planned something like weeks or days ago, I do take a few extra pain killers the previous night or evening to prevent severe headache." T Pg 403<br>" If you keep medicating as much as I do all the time…with triptans too…then you could think that it is less harmful to the body to do only that, than to add yet another thing you should take daily…when I read about side effects, I got really scared and I have not dared to try them…" J Pg 6<br>" I kind of allow myself to take the medication when I feel that I need it, without feeling guilty." J Pg 6<br>"I try to live in some kind of unawareness of how much I medicate…"J Pg 7
Meaningful symptom relief	'…an increase in the number of pain free days would be most meaningful to them.' '….participants indicated a desire for medication that would reliably relieve headache pain.' C Pg 483	Well, I think if you want to measure incremental improvements I would consider it nice just to have a drug [where I could] say, 'OK,I have a headache. I can take this and I'll be OK in an hour." C Pg483
The search for meaning/explanations	'It is the search…for a rational explanation.'L Pg 1623<br>' The representation of the 'disease dimension' comes to light when…the patient finds that a certain medical diagnosis is acceptable and coincides with his/her subjective perceptions.' L Pg 1623	"…I'm scared that something uglier is there but there's nothing…my worry is always the same but when I see that all the tests are negative, then I wonder why I have a headache."L Pg 1623<br>" they diagnosed it as being chronic…daily…all days, there is no day I am free…" L Pg1623

	'They expressed a need to find out what was causing the headache…At times they worried that the headaches were a symptom of serious illness, such as a tumour or a stroke.' J Pg 6	"I used to think that I probably have cancer in the head."J Pg 6

Third order conceptual theme 3 **Strained relationships**

**Aspects**	**Interpretations of: Tenhunen et al 2005, Coeytaux et al 2007, Lonardi 2007, Jonssson et al 2013**	**Quotes/supporting data**
Work	Some 'negative attitudes' when colleagues work life was affected. T Pg 401	"…if I somehow influence their quality of work, they become annoyed." T Pg 401
Friends	'…how their friends responded to their headaches…'T Pg402	"…they don't mind if I cancel something at the last minute…they've got used to it, I think. Or probably they are annoyed but they cope with it…"T Pg 402
	Friends responded to analgesic use differently. T 401Pg	"…if I take a painkiller when I'm visiting them they become worried… or… they try to act as if nothing happened."T Pg401
	'…each group reported strained relationships resulting from the need to change or cancel plans because of headaches.' C Pg 483	"…you have to call people [at the] last minute and say, "I know I committed to this but I can't do it' So… you have to preface every commitment with 'if I can.'" C Pg 483
	'It was hard to disappoint others and be unable to participate.'J Pg 5	"…so I just said 'no, I cannot come' e.g. out to meet friends for birthdays and such. It was really hard." J Pg 5
Changed relationships	'Almost all… reported changes in the way others perceived them.' Seen as weak or put in a sick or dependent role.T Pg401	"…they' ll have a picture of me as a sick girl of the family."T pg402<br>"I used to be the strong one in the family…but now when I'm not so strong people tend to save me from hearing bad news…" T Pg 402
Stress on others	'…headaches placed stress on relationships not only by affecting their own behaviour but also because of the	…"When we are both tired and stressed and I'm in pain and she's worried about me every little thing in our life seems to be magnified." "... she is scared of…if something serious, like lethal, would happen to me." T Pg 402

Helped by others	confusion, frustration and fear experienced by their partners.'T Pg 402<br>‘…their colleagues who were usually understanding and supportive…’ T Pg 401<br>‘The ‘sickness dimension’ which is the social representation of the disease. Scenario 1 The patient is fully accepted into the inner family.’ Sometimes associated with the family being hyper-protective and external risk and harm may be envisaged.L Pg1623	“Most of them, they know about my problem…They usually come to ask me how I am that day and they try and support me.” T Pg 401<br><br>“Fortunately I have a child who helps me in caring about the other children, my husband comes back from the office and then he cooks until the drug takes effect.” L Pg1623
Devalued by others	‘Participants agreed that an important aspect of their headaches involved the tendency of others to discount their impact.’C Pg 493<br>‘…they had been labelled unreliable or accused of malingering. Participants were particularly critical of healthcare professionals…’ C Pg483<br>‘Scenario 2 although the social representation of the disease is managed within the family it is nevertheless incomplete.’ ‘The patient is recognised as having problems due to a disease but this is a reason for constant and explicit under- evaluation. Possibly due to a shared stereotype, everyone has experienced headaches that are generally mild, periodical, and provisional, and go away after taking a tablet.’L Pg 1624<br>Scenario 3 ‘…a person may risk being the victim of social death….experiencing expulsion from the productive world…’<br>Scenario 4 ‘…the inner family network accepts the person as ill but the wider or external network refuses him/her: employers or colleagues can in fact stigmatize the person as unreliable, lazy ,listless while neighbors and friends tend to trivialise the condition.<br>‘Sometimes they sensed that other people were suspicious, presumably thinking that headaches were being used as an excuse.’ J Pg 6	“People don’t understand because you look OK. You look healthy and you’re going on about your everyday [life] trying to get things done…they don’t understand you’re sick.” C Pg 483<br>No data given<br><br>“People who do not have headaches cannot understand. When I married there were quarrels with my husband and relatives because that wife(me) was always moaning, because of her head.”L Pg1623<br><br>“ I tend to isolate myself in the sense that I don’t even want them to ask me how I am…”L Pg 1625<br>“Not totally unappreciated at work, but perhaps not taken into consideration for what I am…I had to go to the emergency room, this is the only way they understand…” L Pg 1625<br><br>“Some people probably think that, “oh my God, she has migraines all the time, is it because he’s lazy and does not want to be here or what?” Yes, it’s like an excuse to stay home or something.” J Pg 6

Appendix 5. Third order saturation of themes table

3rd order themes/subthemes	Tenhunen	Coeytaux	Lonardi	Jonsson
**Headache as a driver of behaviour Direct**				
Prioritisation/changes to lifestyle	X	X		X
Work	X			X
Reduced social contact	X			
Emotional reactions	X			
Loss of control	X		X	X
Inability to plan	X	X	X	X
Measuring headaches		X		
Testing numerous strategies				X
Medication use				X
**Indirect**				
Financial (and work)	X			X
Loss of energy and concentration (and poor sleep)	X			
Poor sleep (and analgesics)	X			
Emotional reactions (and social)	X		X	X
Social activities (and medication)	X			
Increasing medication				X
Link with psychological factors				X
**The spectre of headache**				
Fear of future	X		X	X
Emotional responses	X			X
Potentially affects future relationships	X		X	
Perceived loss of control	X			X
Medication use/beliefs	X			
Meaningful symptom relief		X		
The search for meaning/explanations			X	X
**Strained relationships**				
Work	X			
Friends	X	X		X
Changed relationships	X			
Stress on others	X			
Helped by others	X		X	
Devalued by others		X	X	X
